# From medicine price control to deregulation: assessing policy effects on insulin access in Pakistan’s private pharmacies

**DOI:** 10.1371/journal.pone.0337151

**Published:** 2026-03-20

**Authors:** Amna Saeed, Minghuan Jiang, Najwa Ali Yasin, Jinran Zhao, Caijun Yang, Yu Fang, Zaheer-Ud-Din Babar

**Affiliations:** 1 Department of Pharmacy Administration and Clinical Pharmacy, School of Pharmacy, Xi’an Jiaotong University, Shaanxi, China; 2 Center for Drug Safety and Policy Research, Xian Jiaotong University, Xi’an, Shaanxi, China; 3 College of Pharmacy, Qatar University, Doha, Qatar; Sanmenxia Central Hospital, Henan University of Science and Technilogy, CHINA

## Abstract

**Background:**

Pakistan faces the highest global prevalence of diabetes and has recently implemented a price deregulation policy for medicines not listed on the National Essential Medicines List (NEML). Pakistan’s NEML 2023 is a direct adoption of the 23^rd^ WHO Model EML rather than a country-specific prioritization; nonetheless, it has been used to identify medicines for exemption from price controls.

**Methods:**

This study evaluated the effect of the price deregulation policy on insulin access in Pakistan. We surveyed 30 retail pharmacies across six regions using an adapted WHO/HAI approach. Post-deregulation data on consumer prices and availability were collected. Pre-deregulation prices were extracted from published sources. The insulin prices were standardized to 10 ml at 100 IU/ml. Difference-in-Differences (DiD) analysis was used to compare price changes between non-NEML insulins (treatment group) and NEML-listed insulins (control group), with each insulin product serving as the unit of analysis. The percentage of available insulin products and their affordability for the lowest-paid worker were calculated. Non-parametric tests were performed for categorical analyses.

**Results:**

Overall, the median price of insulins increased by 31.87% (*p* < 0.001) i.e., from PKR 2030 (7.3 USD) to PKR 2678 (9.6 USD), after deregulation, except for short-acting human biosimilar (BS) insulins that stayed stable. As per the DiD-interaction term, prices of non-NEML insulins increased by 4.1 USD (*p* < 0.001) more than NEML insulins after deregulation. The median number of days’ wages for the lowest-paid worker increased from 1.90 days before deregulation to 2.51 days after, to obtain a month’s insulin supply. All surveyed outlets had at least one insulin product available. Originator brands (OB) had higher availability than BS, with 78.0% of human and 48.8% of analogue insulins available as OBs, compared to 51.1% and only 8.3% as BS insulins, respectively.

**Conclusion:**

Insulin prices increased significantly following the deregulation policy, particularly for non-NEML OBs, leading to reduced affordability despite fair availability. A policy review and stronger financial protection measures are needed to ensure equitable access to insulin.

## 1. Introduction

Diabetes is one of the fastest-growing conditions and has emerged as a major global health challenge in the past few decades. According to the International Diabetes Federation (IDF) Atlas 2025, approximately 589 million adults are living with diabetes, a number expected to increase to 853 million by 2050. Notably, 81% of this diabetic population resides in low- and middle-income countries (LMICs) [1]. Pakistan, an LMIC, is reported to have the highest age-standardized diabetes prevalence rate, i.e., 31.4%. 0.23 million people are estimated to die in Pakistan due to diabetes. Notably, 21.9% of the Pakistani population is living below the poverty line, and 47.3% of the healthcare expenditure is paid out of pocket (OOP) [2,3]. In this context, access to affordable medicines to manage diabetes is crucial for Pakistan.

Insulin is a life-saving medicine for people with insulin-dependent diabetes (Type I), and is essential to manage advanced non-insulin-dependent diabetes (Type 2) or other forms of diabetes to maintain glycemic control and avoid complications. Even a century after its first clinical application, insulin remains central to the lives of many people with diabetes [4]. So far, several insulin products have been developed and marketed, including porcine, human, and analogue insulins, with varying durations of action, ranging from short to ultra-long acting [5].

Despite the wide range of insulin products, inadequate access continues to place people with diabetes at risk of severe complications, including vision loss, amputations, renal failure, cardiovascular disease, and premature mortality [1,6]. Although insulin is off-patent and included in the WHO Model List of Essential Medicines, access remains poor globally, particularly in LMICs. A multi-country study across 13 LMICs reported insulin availability below 80% in both public and private sectors, with neither human nor analogue insulin affordable for the lowest-paid unskilled government worker [7].

In Pakistan, medicines are offered free of charge in the public sector hospitals. However, insulin is often restricted to inpatients and not available to outpatients in these facilities. Hence, the majority of diabetic patients turn to private pharmacies, where they pay OOP to obtain it. This results in a significant financial burden, particularly for those with type 1 diabetes who need an uninterrupted supply of insulin for the rest of their lives. Although insulin is listed on NEML, public-sector procurement is constrained by limited health financing. Whereas, Pakistan’s diabetes-related health expenditure remains among the lowest globally (79 USD per capita) [8]. In 2022, a national survey of public and private medicine outlets in Pakistan found that no insulin product met the WHO benchmark of 80% availability, and all insulins were unaffordable for low-income individuals [9].

The insulin market is distinct from other medicines due to its high level of market concentration, with three multinational companies (MNCs) dominating global and local supply, primarily through originator products, constraining competition and contributing to persistently high prices in Pakistan and other LMICs(7,8).

In recent years, the Drug Regulatory Authority of Pakistan (DRAP) has implemented several medicine pricing mechanisms to improve access by controlling prices, most notably the National Drug Pricing Policies of 2015 and 2018 [10]. However, in February 2024, the DRAP introduced a significant shift by announcing a price deregulation policy for all medicines not listed on the NEML [11]. This marked a departure from decades of centralized control of the retail prices of medicines in Pakistan, where all medicines were not allowed to be sold to consumers beyond a fixed retail price set by the regulator. Under the deregulation policy, manufacturers were permitted to set and revise retail prices of non-NEML medicines without prior regulatory approval, with the expectation that increased pricing flexibility would encourage competition, improve supply, and reduce shortages.

Despite these intentions, concerns have emerged regarding price increases in therapeutic classes characterized by limited competition and low local production. These concerns are amplified by the fact that the deregulation criterion relies solely on the NEML, which is largely a direct adoption of the WHO Model EML and not adapted to Pakistan’s country-specific disease burden or market realities [12]. As a result, several widely used medicines for chronic conditions, including insulins, fall outside price protection mechanisms. In the absence of complementary safeguards, such as pricing oversight, targeted subsidies, or financing mechanisms, already unaffordable medicines may experience disproportionate price increases, particularly in concentrated markets with scarce local manufacturing capacity, as is the case with insulin in Pakistan [9]. Consistent with this, prior research has shown that LMICs such as Pakistan often lack a systematic approach to medicine price regulations, limiting the effectiveness of policy interventions despite extensive documentation of pricing challenges [13–15].

In light of these concerns, monitoring the impact of recent pricing deregulation in Pakistan on access to insulin is essential. Thus, this study compares the prices and affordability of insulin products before and after the deregulation policy, highlighting the differences between those that are and are not on the NEML. Besides providing a national scale of access to all insulin products in terms of their prices, availability, and affordability, this study offers the first empirical evidence on the consequences of this shift in medicine pricing policy on insulin access in the country.

## 2. Methods

### 2.1. Study design

This cross-sectional survey was carried out using an adapted WHO/Health Action International (HAI) methodology. All available insulin products were surveyed in private retail pharmacies across Pakistan.

### 2.2. Sampling of survey areas

**Fig 1:**
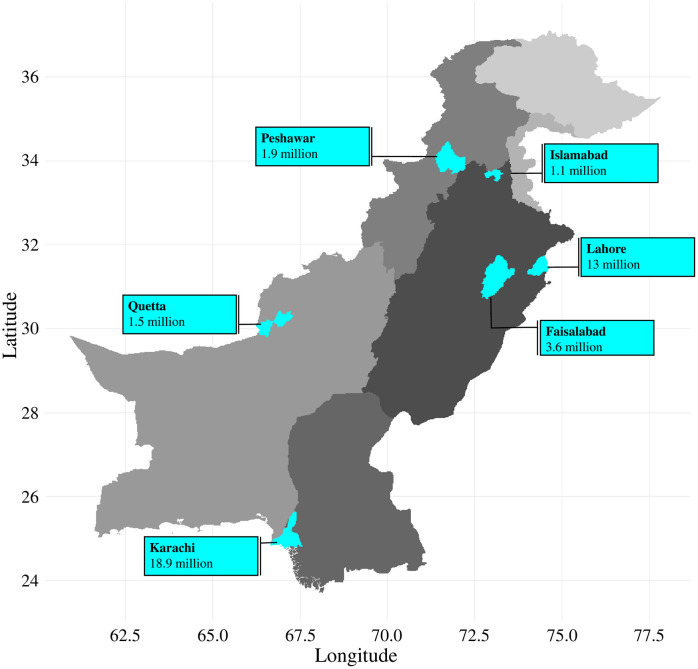
Geographic distribution of survey areas across Pakistan. Grey shading represents provincial population gradients, while cyan areas indicate surveyed cities. Numbers adjacent to each city indicate population size. The map was created by the authors using R (version 4.4.1) with the *sf* and *ggplot2* packages, based on administrative boundary data from the Global Administrative Areas database (GADM; https://gadm.org). The figure is an original work and is published under the Creative Commons Attribution (CC BY 4.0) license.

According to WHO/HAI methodology, at least six survey areas must be selected within the main survey region or country, corresponding to government-defined administrative units (e.g., cities or districts), to ensure adequate geographical representation [16]. In this study, Pakistan was treated as the main survey region, and six major cities were selected as survey areas, ensuring representation from each province as well as the federal capital. These survey areas included Islamabad (the federal capital), Lahore (the provincial capital of Punjab), Faisalabad (a major city in Punjab), Peshawar (the provincial capital of Khyber Pakhtunkhwa), Karachi (the provincial capital of Sindh), and Quetta (the provincial capital of Baluchistan). Punjab, being the most populous province, was represented by two survey areas (See Fig 1). This selection ensured that at least one survey area was included from each province and that the federal capital was represented separately, thereby maintaining national-level representativeness in accordance with WHO/HAI guidance.

### 2.3. Sampling of survey units

Private sector retail pharmacies were selected from the survey areas following the WHO/HAI-based sampling approach. According to standard WHO/HAI methodology, the largest public sector hospital in each survey area serves as the survey anchor, and surrounding medicine outlets are selected based on their proximity to this anchor. A list of all public sector hospitals was obtained from the official website of the Pakistan government health department. From this list, the largest tertiary-care public hospital based on its number of beds was selected as the primary anchor in each survey area. Subsequently, four additional public-sector hospitals within a three-hour travel radius of the anchor were identified to delineate the survey catchment area.

Within the vicinity of each of these hospitals, five registered private retail pharmacies located within a 10-kilometer radius were selected. This purposive, distance-based sampling technique, recommended by WHO/HAI, was applied consistently across all six survey areas. Data were collected exclusively from registered private retail pharmacies for three reasons: [1] In Pakistan, most of the non-NEML insulins are only provided at retail pharmacies, [2] the insulins are provided free of charge to the patients in public sector hospital pharmacies, and [3] Outpatient availability of insulin in public facilities is limited, leading most patients to rely on private pharmacies where they pay out-of-pocket (OOP) [9]. Similar surveys targeting retail pharmacies and employing a variant of the WHO/HAI methodology have been performed previously [17]. In total, 30 private retail pharmacies (five per survey area) were included in the study.

### 2.4. Data collection and entry

Price and availability data for all insulin products found at each pharmacy were collected using a standardized, paper-based data collection form during June–July 2024. Subsequently, each product was categorized by: Duration of action (short-, intermediate-, long-acting insulin); Origin (human or analogue insulin); Product type (originator or biosimilar); Strength; and Presentation (vial, cartridge, or pre-filled injections). Whereas, the OB is the first product that received market authorization, and biosimilar (BS) is a biologic product that has a high similarity with OB.

Data were collected by final-year pharmacy students, who received structured training from the first author based on the WHO/HAI methodology [16]. The data collection form was pilot-tested at two big retail pharmacies before the final data collection. The data collectors visited the survey units and noted the availability of each insulin product after physically checking the stock. They also noted Maximum Retail Prices (MRP) per pack from the insulin packages. Prices for 10 ml of insulins were also calculated and entered in the data collection form. The data were validated by re-collecting the data in one retail pharmacy per survey area. To enable a comparison between current (post-deregulation) prices of all available insulin products in the Pakistani market and their pre-deregulation prices, historical price data were obtained for each product found in the post-deregulation survey from the Pharma Guide, 31st edition (2024). The Pharma Guide’s most recent DRAP-regulated prices are shown in this edition as of December 1, 2023, just before non-NEML medications were deregulated [18]. The Pharma Guide is widely recognized as a trustworthy public source on medication pricing and has been used in many studies to establish baseline prices, making it a reliable resource for retrospective analyses [19–21].

The post-deregulation availability data of insulin products, and the prices (before and after deregulation) data of each insulin product were entered into Excel spreadsheets. The entered data was then double-checked, cleaned, and consolidated to ensure accuracy and minimize mistakes. The manufacturer’s website was also consulted to verify the types, brands, and amounts of insulin used in each presentation. The dataset underlying the analyses is provided as S1 Dataset in the Supporting Information.

### 2.5. Data analysis

#### 2.5.1. Prices.

The prices of insulins in Pakistani Rupee (PKR), were standardized to 10 ml of 100 IU/ml. The local prices were converted to US dollars using the OANDA currency converter at the exchange rate on the first day of data collection [22]. The median and interquartile range (IQR) for insulin prices were calculated. Insulin prices were compared among different categories, i.e., NEML status (NEML vs non-NEML), origin (human vs analogue), product type (OB vs BS), presentation (vials, cartridges, and pre-filled pens), before and after deregulation. The significance of price changes among insulin products following deregulation was calculated by running Wilcoxon signed-rank tests. Statistical significance was determined at a *p*-value threshold of 0.05. Given the six-month interval between pre- and post-deregulation prices, overall price trends were examined first to characterize market-level changes. Subsequently, differential effects by NEML status were evaluated using the Difference-in-Differences (DiD) regression.

#### 2.5.2. Impact of price deregulation on insulin prices.

DiD analysis, a quasi-experimental technique, was employed to analyze the effects of price deregulation policy on insulin prices in Pakistan. It is a well-established method used for health policy evaluations [23,24]. The analysis compared insulin price changes in NEML versus non-NEML groups, where the non-NEML insulins served as the treatment group, as the deregulation policy is focused on medicines in this category only. The insulins enlisted on the NEML were considered the control group.

The primary outcome variable was standardized insulin prices (10 ml) in PKR. The unit of analysis was the individual insulin product observed at two time points (pre- and post-deregulation). The key explanatory variables were NEML status (NEML vs non-NEML insulins) and time period (pre- vs post-deregulation). The covariates included duration of action (short, intermediate, and long-acting insulin), origin (human and analogue), product type (OB and BS), and presentation (vial, cartridge, and pre-filled pens).

Baseline DiD model without confounders:


$$Y_{it}=\beta_{\text{0}}+{\beta_{\text{1}}neml_{i}+\beta }_{\text{2}}dere{gulation}_{t}+\beta_{\text{3}}\left(neml_{i}\times deregulation_{t}\right)+\varepsilon_{it}$$

Where, $Y_{it}$ is the outcome variable (insulin price) for the product *i* at time *t.,* $neml_{i}$ is a dummy variable indicating whether the product is non-NEML or NEML, $dere{gulation}_{t}\;$is a dummy variable indicating whether the observation is post deregulation, $\beta_{\text{3}}$ represents the DiD estimator, capturing the differential effect of deregulation on non-NEML products compared to NEML products.

Extended model with confounders:


$$Y_{it}=\beta_{\text{0}}+\beta_{\text{1}}neml_{i}+\beta_{\text{2}}deregulation_{t}+\beta_{\text{3}}\left(neml_{i}\times deregulation_{t}\right)+\beta_{\text{4}}X_{it}+\varepsilon_{it}$$

Where $X_{it}\;$presents a vector of control variables, i.e., product type, origin, and presentation type etc.

To account for heteroskedasticity, robust standard errors were used to estimate both models.

#### 2.5.3. Affordability.

The affordability of insulins was expressed as the number of days’ wages (NDWs) required for a lowest-paid government employee to buy the 10 ml insulin, i.e., approximately a month’s supply. The salary was taken as 32000PKR per month (with effect from July 2023) [25]. According to the WHO/HAI benchmark, if a patient has to spend more than 1 day’s wage for treatment with a specific medicine in a month, that medicine is considered unaffordable.

#### 2.5.4. Availability.

The availability was measured as percentage availability, considering the number of retail pharmacies with a particular insulin product available on the day of data collection. The following is a description of the % availability: absent, meaning that at the time of the survey, none of the surveyed pharmacies stocked the specific insulin product; fairly low, meaning that the insulin product was present in less than 30% of facilities; low, meaning that it was present in fewer than 50% of facilities; fairly high, meaning that between 50% and 80% of facilities had the insulin product; high, meaning that the majority of the facilities had the insulin [26]. The availability analysis consisted of data from the post-implementation period of the deregulation policy.

These analyses were performed using Microsoft Excel and Stata (version 15; StataCorp LLC, College Station, TX, USA). All descriptive results were presented following standard WHO/HAI reporting categories to maintain consistency with international medicine price and availability surveys.

### 2.6. Ethical approval and consent to participate

Ethical approval was obtained from the ethical review committee of the pharmacy practice department, Bahauddin Zakariya University, Multan, Pakistan (Letter no. BZU-FOPDPP-2455). The study did not involve patients or the collection of personal or identifiable data; only publicly available maximum retail prices of insulin products were collected from sampled private retail pharmacies. Verbal informed consent was obtained from the pharmacists or pharmacy managers before data collection.

### 2.7. Inclusivity in global research

Additional information regarding the ethical, cultural, and scientific considerations specific to inclusivity in global research is included in the Supporting Information (S1 Checklist).

## 3. Results

### 3.1. Insulin prices by product characteristics

The median insulin price (per 10 mL) increased significantly following deregulation, rising from PKR 2030 (7.3 USD) to 2678 (9.6 USD), representing a 31.9% increase (Wilcoxon signed-rank test, *p* < 0.001). By NEML-enlistment status, NEML insulins were consistently priced lower than non-NEML insulins both before (PKR 1035 vs. 3367) and after deregulation (PKR 1267 vs. 4622); however, post-deregulation price increases were larger among non-NEML insulins (37.2%) than NEML-listed insulins (22.2%) (*p* < 0.001) (Table 1).

**Table 1:** Median prices (10 mL 100 IU/mL) of insulins by presentation type, and product category pre- and post-deregulation.

Category	N	Median price (PKR) Pre (IQR)	Median price (PKR) Post (IQR)	Median price Pre (USD)	Median price Post (USD)	Percent change	Wilcoxon z-value	*p-*value	Effect size (r)
Overall	380	2030.0 (975.0-4288.6)	2678.0 (1196.8-5336.6)	7.3	9.6	31.8%	−15.8	<0.001	−0.8
**NEML enlistment status**									
NEML	140	1035 (916–1035)	1267 (1096.8–1267)	3.7	4.5	22.25%	−9.8	<0.001	−0.8
Non-NEML	204	3367.3 (2030-4288.6)	4622 (2678-5336.7)	12.1	16.64	37.18%	−12.3	<0.001	−0.9
**Origin**									
Human	184	975.0(910.5-1210.8)	1230.5(916.0-1303.0)	3.5	4.4	26.2%	−10.7	<0.001	−0.8
Analogue	196	4288.6 (3746.6-4717.4)	5336.6(4626.6-5586.5)	15.4	19.2	24.4%	−11.4	<0.001	−0.8
**Product type**									
Originator	300	3746.6 (1241.0-4396.6)	4626.6 (1267.0-5377.3)	13.5	16.6	37.2%	−14.6	<0.001	−0.8
Biosimilar/Generic	80	900.0 (840.0-916.0)	900.0 (900.0-1303.0)	3.2	3.2	0%	−4.8	<0.001	−0.5
**Presentation**									
Vial	168	975.0 (900.0-1035.0)	1096.8 (916.0-1267.0)	3.5		19.7%	−10.2	<0.001	−0.8
cartridge	77	3246.6 (1666.6-3746.6)	4622.0 (1856.6-4798.6)	11.7	16.6	24.6%	−7.3	<0.001	−0.8
Pre-filled pen	135	4396.6(4288.6-4717.4)	5342.0(5146.0–6080.0)	15.8	19.2	42.2%	−9.3	<0.001	−0.8

**Fig 2:**
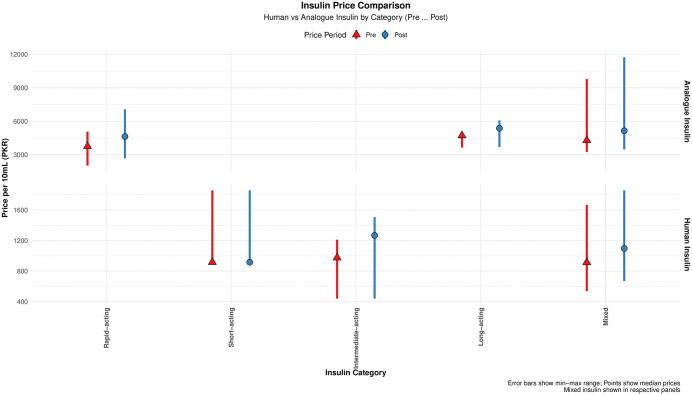
Prices of human versus analogue insulins before and after deregulation across therapeutic categories.

When stratified by origin, analogue insulins were consistently priced higher than human insulins both before and after deregulation. Median prices of human insulins increased from PKR 975.0 to 1230.5, while analogue insulin prices rose from PKR 4288.6 to 5336.6 (*p* < 0.001) (Fig 2).

In terms of duration of action, the intermediate-acting human insulins experienced the highest price hike, by 30%. Among the insulin analogues, Lispro (a rapid-acting analogue) experienced the highest price increase of 28%, rising from PKR 3367.30 to 4622.0 post deregulation (S1 Table).

Product-wise, OBs were priced substantially higher than BS both before and after deregulation and experienced a marked price increase from PKR 3746.6 to 4626.6 (*p* < 0.001). In contrast, BS showed no change in median price, remaining at PKR 900.

Among presentation types, prefilled pens were priced higher than cartridges and vials at both time points, increasing from PKR 4396.6 to 5342.0, compared with cartridges (PKR 3246.6 to 4622.0) and vials (PKR 975.0 to 1096.8). The prefilled pens exhibited the highest price increase (42.2%), followed by cartridges (24.6%) and vials (19.7%).

### 3.2. Differential effect of deregulation on insulin prices: DiD analysis

Table 2 presents the findings from the DiD analysis. Both models showed significant price hikes among non-NEML insulins post-deregulation. In model 1 (without confounders), the DiD interaction term showed a differential increase of PKR 998 (p < 0.001), which further increased to PKR 1142 (p < 0.001) after adjusting for insulin characteristics, indicating that non-NEML products were disproportionately impacted by the policy.

**Table 2:** Difference-in-differences regression results for insulin prices.

Variables	Model 1 Without Confounders	Model 2 With Confounders
**Period (Post vs. pre deregulation)**	1379.2 (223.3)***	1379.2 (208.8)***
**NEML status (NEML vs. non-NEML)**	−2509.1 (178.3)***	−206.19(146.7)
**DiD (period × NEML status)**	−997.9 (268.9)***	−1141.5 (211.4)***
**Origin (Analog vs. Human Insulin)**	–	3131.6 (196.7)***
**Product type (Biosimilar vs. Originator)**	–	−374.8 (45.1)***
**Duration of Action (Long vs. intermediate vs. short vs. rapid acting)**	–	202.0 (35.3) ***
**Presentation (Prefilled pens vs. cartridges vs. vials)**	–	257.8 (117.1)*
**Constant**	4346.97 (146.8)***	2671.75 (189.7)***
**Observations (N)**	760	760
**R-Squared**	0.4	0.8
**F-statistic**	175.8	1373.7
**Root MSE**	1692.7	1016.5

Robust standard errors are reported in parentheses. *Significance levels: ***p < 0.001, **p < 0.01, *p < 0.05

Furthermore, both models confirmed that the prices of insulins were significantly higher overall after deregulation by PKR 1379.2 (*p* < 0.001) than they were before.

The baseline price gap between NEML and non-NEML insulins (PKR –2509; p < 0.001) became statistically insignificant after adjustment, reflecting that observed differences were largely explained by product attributes.

Among covariates in the adjusted model, OBs were more expensive than biosimilars (PKR 374.8; *p* < 0.001), analogue insulins were markedly costlier than human insulins (PKR 3131.6; *p* < 0.001), and prefilled pens were more expensive than vials (PKR 257.8; *p* < 0.05). The adjusted model demonstrated substantially improved model fit (R² = 0.80 vs. 0.40), indicating that accounting for insulin characteristics greatly increased explanatory power.

### 3.3. Affordability

After deregulation, the affordability was significantly reduced, particularly for OB analogue insulins. Within six months of the deregulation policy’s launch, the median NDWs increased from 1.9 days before deregulation to 2.5 days after it, a 32% decline in the overall affordability of insulins (Table 3).

**Table 3:** Insulin affordability by origin, presentation type, and NEML enlistment status pre- and post-price deregulation.

Category	N	Affordability (NDWs) Pre	Affordability (NDWs) Post	% Change
**All products**	**380**	**1.9**	**2.5**	**32%**
By product type				
Originator	301	3.2	4.3	37%
Biosimilar/Generic	79	0.8	0.8	0%
**By Origin**				
Human	184	0.9	1.1	26%
Analogue	196	4.0	5.0	25%
**By presentation**				
Vial	168	0.9	1.0	20%
pre-filled pen	135	4.0	5.0	25%
Cartridge	77	3.0	4.3	42%

NDWs: Number of days’ wages required by a low-paid, unskilled government worker to purchase 10 ml of 100 IU insulin (approximate monthly treatment course).

Only the BS insulins remained affordable both before and after deregulation, with the NDWs remaining static at 0.8. Contrarily, the OB insulins remained unaffordable at both time points, showing a 73% decrease in affordability.

In the human versus analogue comparison of affordability, human insulins remained marginally affordable; however, they did show a reduction in affordability by 26%. On the other hand, the analogue insulins remained unaffordable pre- and post-deregulation, with 4 and 5 NDWs, respectively, a reduction in affordability by 25%. Among analogue insulins, particularly the long-acting analogues of OB, such as detemir and glargine, highly unaffordable levels were observed, exceeding 5 days’ wages per 10 ml. Notably, the mixed analogue formulation, i.e., Aspart/Degludec was the least affordable with more than 10 NDWs post-deregulation. Among the specific insulin products, OBs of intermediate-acting human insulin and Lispro (a rapid acting insulin analogue) showed a substantial reduction in affordability, i.e., by 30% and 37%, respectively (S2 Table).

When stratified by presentation type, although all types experienced an increase in the NDWs, vials remained relatively affordable in both periods, i.e., 0.9 and 1.0 NDWs. Meanwhile, the prefilled pens and cartridges remained unaffordable at both time points, resulting in a 25% and 42% reduction in affordability.

### 3.4. Post-deregulation availability of insulin

**Fig 3:**
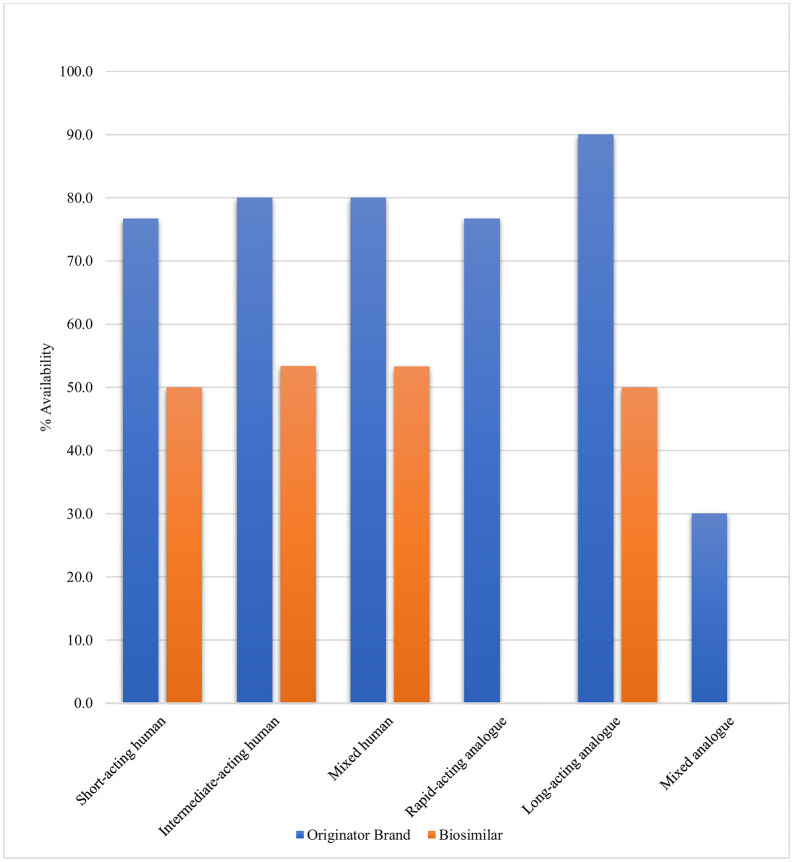
Availability of originator and biosimilar insulins by duration of action and origin.

Overall, OB insulins were more widely available than BS, with 78.0% of human and 48.8% of analogue insulins available as OBs, compared to 51.1% and only 8.3% as BS insulins, respectively. This trend highlights how OBs continue to dominate the market, even for insulin types with BS substitutes (Fig 3).

OB Glargine, a long-acting insulin analogue, was found to be the highest available insulin type (90%) (S3 Table). Besides glargine, only OBs of isophane and 70/30 (isophane/regular) had the ideal availability, i.e., 80%. OB glulisine had very low availability (<30%), while degludec was not found in the market. BS products of insulin regular, isophane, 70/30 (isophane/regular), and glargine were found to have fairly high availability of 50%, 53.3%, and 50%, respectively (Table 4).

**Table 4:** Product-wise availability of insulins.

Availability	Originator brands	Biosimilar/Generics
Insulin not found in any outlets (0%)	Degludec	Aspart, Glulisine, Lispro, Degludec, Detemir, Mixed Analogues
Insulin with very low availability (<30%)	Glulisine	
Insulin with low availability (30–49%)	Degludec/Aspart, Detemir, Lispro	
Insulin with fairly high availability (50–80%)	Isophane, Regular, Aspart, Iso/reg-70/30	Regular, Isophane, Glargine, 70/30 (Isophane/Regular)
Insulin with high availability (>80%)	Glargine	

### 3.5. Market composition of insulin products

A total of 380 insulin products (including duplicates) were found in the private sector market (Table 5). 48% (n = 184) of these were human insulin products and 52% (n = 196) were analogue insulins. 85% of these products was manufactured by three MNCs i.e., Eli Lilly (39%), Novo Nordisk (34%), and Sanofi Aventis (12%). However, only 15% of these products were manufactured collectively by local manufacturers. The share of available insulin products by presentation type was Vial>Prefilled Pen>Cartridge. The NEML insulins (65%) were relatively more available than non-NEML enlisted insulins (35%).

**Table 5:** Number of insulin products found in private pharmacies by type, manufacturer, and NEML enlistment.

Characteristics		Insulin products %(n)
Type of insulin		
	**Human insulin**	48% (184)
	Short-acting human	14% (52)
	Intermediate-acting human	14% (53)
	Mixed human	21% (79)
	**Analogue insulin**	52% (196)
	Rapid-acting analogue	32% (120)
	Long-acting analogue	18% (67)
	Mixed analogue	2% (9)
Manufacturer		
	Eli Lilly	39% (148)
	Novo Nordisk	34% (129)
	Sanofi Aventis	12% (44)
	Local Manufacturers	15% (59)
Presentation		
	Vial	44% (168)
	Prefilled Pen	36% (135)
	Cartridge	20% (77)
NEML-enlistment		
	Listed on NEML	65% (250)
	Not Listed on NEML	35% (130)

### 3.6. City-wise average number of insulin products per pharmacy

**Fig 4:**
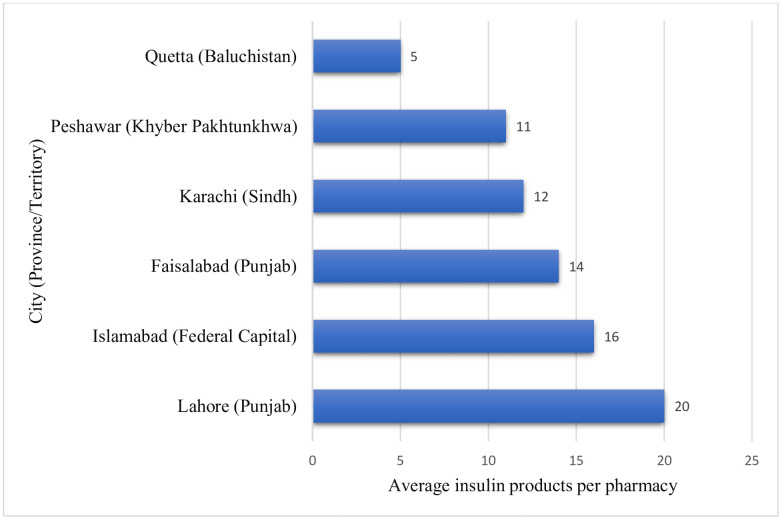
City-wise average number of insulin products per retail pharmacy.

All surveyed outlets had at least one insulin product available. However, the average number of products available at retail pharmacies varied across different regions and ranged from 5 in Quetta (Baluchistan) to 20 in Lahore (Punjab)(S4 Table and Fig 4).

## 4. Discussion

To the best of our knowledge, this is the first national study to empirically assess the impact of Pakistan’s recent medicine price deregulation policy on insulin prices and affordability using a quasi-experimental approach. Additionally, it presents national-scale insights into insulin access in private retail pharmacies across the country. After deregulation, the prices of nearly all categories of insulin experienced a significant hike, particularly in OBs, analogues, and prefilled pens. The overall insulin price increase of 32% post-deregulation in an LMIC like Pakistan is particularly concerning [2,3]. Notably, about 85% of all medical expenses in Pakistan are spent at private facilities, indicating the country’s heavy reliance on the private sector [27]. Thus, the current study—which focuses on insulin access in private sector settings—is particularly relevant and crucial for guiding evidence-based policy and enhancing equitable access to these essential diabetes treatments. Although BS human insulins did not exhibit price increases, their market availability was comparatively limited. While the overall affordability of insulin for those on low wages was found to be poor, both before and after deregulation, it was reported to have been significantly reduced further within just six months of the policy’s launch. However, insulin availability was found to be moderate to high for most insulin products post-deregulation.

### 4.1. Insulin prices by product characteristics

The analogue insulins were at substantially higher prices than human insulins at both time points. This finding aligns with the observations from not only LMICs but also HICs(7). For instance, the prices of insulin analogues have been reported to be ten times higher than those of regular or NPH human insulins in the US [28]. Before deregulation, the OB prices were approximately 4.2 times higher than BS and increased to 5.2 times higher after deregulation. A previous study conducted in 2019 reported this difference to be much less in Pakistan, i.e., OBs were priced 1.34 times higher than BS insulins [9]. This widening price gap suggests increasing potential for cost savings through greater use of BS insulins.

Notably, price increases were observed primarily among OBs, while BS prices remained stable. The static and lower prices of BS products may reflect competitive pressures from MNCs and limited market share, as observed in several other LMICs [7, 29]. Among the human insulins, the intermediate-acting insulins, i.e., Isophane (NPH), showed the highest price increment. This finding is particularly concerning as NPH is not only part of the NEML but also one of the most frequently prescribed insulins globally and is considered an affordable option for cost-sensitive patients [30]. Among the analogue insulins, Lispro underwent the largest price increase (28%), followed by Aspart and Glargine. Although all analogues were high-priced, the relatively lower price increment in glargine may be partly attributable to the presence of its BS product in the market. A study conducted in 28 European countries reported a significant price reduction for the originator glargine after the entry of BS glargine in the markets [31]. Among the various presentations, prefilled pens experienced the highest price hike following deregulation. Although the use of prefilled insulin pens is comparatively less prevalent in LMICs, their high prices have caused great concern in developed countries where their use is frequent [32].

### 4.2. Impact of deregulation on insulin prices: insights from Difference-in-Differences analysis

The findings from the adjusted DiD model revealed that the prices of non-NEML insulins increased disproportionately compared to those listed on the NEML. This may be attributed to the poor market competition for insulin in Pakistan, where a few MNCs, like several other LMICs(7). Importantly, the DiD estimates isolate the differential price change associated with deregulation after accounting for baseline differences and product characteristics, strengthening the interpretation that non-NEML insulins were more strongly affected by the policy change. The deregulation might have enabled oligopolistic pricing practices for this already high-priced medicine, further restricting its affordability. A study conducted in China to gauge the effect of deregulation policy on drug prices found similar results, stating that price changes after policy implementation varied among drug categories [33]. It is also noteworthy that although the price deregulation was employed for the non-NEML medicines, the prices of NEML insulins, particularly OBs, were also increased. Liu et al. also found that the cost of essential medications had increased significantly, affecting low-income groups [34], following the implementation of the medicine price deregulation. This suggests that price deregulation, even if aimed at non-NEML medications, may have wider market effects and result in unexpected price hikes for essential medicines as well. The persistently poor availability of BS products in the market would be a great challenge in realizing these savings. In the unadjusted model, NEML insulins were significantly cheaper than non-NEML insulins. After accounting for product characteristics, the difference became statistically insignificant. This suggests that the lower prices of NEML insulins are largely attributable to the types of products listed on the NEML rather than the NEML inclusion itself.

### 4.3. Insulin affordability

Despite differences across product types, affordability remained poor for most insulin categories both before and after deregulation, with a further deterioration observed after the policy. This indicates that price increases directly translated into increased financial burden for patients, particularly those dependent on OBs and analogue insulins. These results are in line with previous evidence from Pakistan, where a 2019 study indicated these products to be unaffordable. Similar findings have also been reported in two states of India, where Sharma et al. and Satheesh et al. documented comparable challenges in the private sector [35, 36]. Notably, both Pakistan and India still struggle with low affordability, even though India has more domestic insulin production capacity than Pakistan. This shows that local manufacturing may not be enough to guarantee access to affordable BS insulin products, particularly when MNC products are more frequently stocked and preferred by patients and providers [36]. Only the human BS insulin remained marginally affordable, i.e., NDW < 1, at both time points. This finding is opposite to the one in our previous study conducted five years ago, where all insulin products were found unaffordable (NDW > 1), including human BS insulins. This suggests that human BS insulin has become slightly more affordable over time, either as a result of improved minimum wages, price changes, or heightened market competition.

### 4.4. Insulin availability

As availability data were collected only post-deregulation, the findings in this section describe current market availability rather than the causal effects of the deregulation policy. Pakistan’s insulin market is dominated by a few MNCs exerting significant market control. Our data indicated that local manufacturers predominantly produce human insulin formulations, regular insulin, isophane (NPH), and premixed isophane/regular (70/30), mainly supplied as 100 IU/mL vials, while production of insulin analogues is limited to a single long-acting analogue (glargine), largely available as prefilled pens; moreover, domestic production is concentrated among a small number of firms, suggesting limited diversification into analogue and combination products. After deregulation, the overall insulin availability in private pharmacies of Pakistan was found to be moderate to high. Notably, only OBs of Glargine and isophane/regular (70/30) had availability above the WHO’s benchmark of 80%. This finding was contrary to findings from our study in 2019, where none of the insulin products had availability above 80% [9]. Although this suggests improvement in the availability of these products over time, this change cannot be directly associated with deregulation policy, as city-level availability data were not collected in the current study. However, recent news reports and interviews with representatives of the Pakistan Pharmaceutical Manufacturers’ Association suggest that deregulation has improved drug availability, with a reported 21.7% growth in market value driven by new launches, line extensions, and the return of hardship products [37]. Though the pharmaceutical manufacturing businesses may have benefited overall from this policy, there is no funding or policy to preserve patients’ affordability, particularly for those with low incomes, for expensive and frequently prescribed medications to treat highly prevalent diseases such as diabetes. Besides, clarifying the causal relationship between policy changes and insulin availability will require future longitudinal research with more detailed data.

It appears that Pakistan’s insulin market, like those in several developing countries, is witnessing a shift from human insulin to analogue insulin, as the only insulin product with the highest availability (90%) was OB of a long-acting insulin analogue glargine [36,38,39]. Whereas in 2019, none of the analogue insulins had availability beyond 80%. The availability of BS glargine also improved from <30% to 50%. Similarly, the availability of OBs of insulin aspart and detemir also improved. On the other hand, compared to 2019, the availability of human insulin products stayed somewhat similar. The increasing use of analogue insulin formulations is possibly the reason for the rise in their availability in Pakistan’s private sector between 2019 and 2024 [9]. Notably, the clinical efficacy of costlier insulin analogues, which increase the financial burden on healthcare systems and patients, is still debated [40]. Analog insulin helps lower hypoglycemic episodes and weight gain, improve treatment adherence, lessen anxiety over dose adjustments, and increase patient satisfaction, according to evidence primarily from higher-income nations [25,27]. Conversely, only 23% of the 64 comparison trials reviewed indicated that analogues were more effective at reducing A1C levels [41]. Therefore, the growing availability of analogue insulins in Pakistan poses significant concerns regarding cost-effectiveness and equitable access in a healthcare system with limited resources, even as it reflects changing clinical preferences and market realities.

Another notable shift in the insulin availability over the past five years was the decline in the availability of vial formulations and the increase in the availability of high-cost prefilled pens, similar to those in several other countries [9,36,39]. In 2019, the vials accounted for 58.4% of all insulin products in the market, which dropped to 44% by 2024. Conversely, the percentage of prefilled pens rose from 29.6% to 36%, indicating a steady shift toward more convenient but more costly delivery devices [9]. Since analogue insulin pens and cartridges are more costly than human insulin vials, such a shift will have a significant financial impact on the local healthcare system. As per our findings, the lowest-paid unskilled worker in Pakistan would have to pay 1.03 days’ wage to get the vial formulations compared to 5.04 days’ wage to get the prefilled pen. Furthermore, patients with diabetes pay considerably more to address comorbidities.

We found that all surveyed pharmacies across six cities had at least one insulin product available, indicating a baseline level of market presence. However, possible regional disparities in insulin accessibility were highlighted by the noteworthy variations in the average number of stocked insulin products, ranging from 5 in Quetta (Baluchistan) to 20 in Lahore (Punjab). These discrepancies reflect geographical variations in insulin demand, economic development, and health infrastructure. The availability was significantly higher in cities like Lahore and Islamabad, which are located in more developed provinces with better health systems, higher health budgets, and more affluent populations [42]. Quetta’s restricted access, on the other hand, might be due to a smaller population size and underfunding of Baluchistan’s healthcare system, which is in line with the region’s lower Human Development Index and subpar healthcare delivery [43]. On the other hand, insulin availability varied among pharmacies in major cities such as Karachi, a sign of inefficient supply chains and irregular stocking procedures. These results suggest that region-specific efforts and a fairer distribution method are needed to enhance insulin access across the country.

### 4.5. Policy implications

While multiple factors may have influenced insulin price changes over the past two years, our findings indicate that the deregulation policy was associated with disproportionately higher price increases for non-NEML insulins. The finding that non-NEML insulins experienced larger price increases than NEML-listed products underscore the importance of reassessing the use of NEML status as the sole criterion for price deregulation. A more nuanced approach could be considered by policymakers, especially for diabetes treatments like insulin that are supplied through a highly concentrated market.

The stable prices of BS insulins signify their competitive potential. Therefore, there is a dire need to promote market competition by supporting the local insulin manufacturers through targeted interventions such as investment in local production, pricing incentives, technology transfer, and a streamlined registration process.

In order to build public trust and encourage the acceptance of locally produced BS insulins, their efficacy and safety must be compared with those of MNCs, and results must be shared and promoted publicly. Literature shows that the entry of BS insulins has led to significant price reductions of expensive analogues such as glargine [31], and the efficacy of these products is comparable [44]. Pakistani insulin manufacturers partner with the government to promote the public health advantages of less expensive human insulin and biosimilars as part of their corporate social duty.

The amplified use of insulin analogues and prefilled pens, despite limited evidence on superior efficacy, emphasizes the need for the implementation of policy tools such as prescription evaluations and health technology assessment to guide the cost-effective use. The use of analogues must also be restricted by devising and implementing evidence-based guidelines prioritizing the use of less expensive and quality-assured human insulins.

After deregulation, the spillover effects, evident from the indirect price increases observed in NEML insulins, although lower than those of non-NEML products, highlight the need for a strong and continuous medicine price monitoring system. There is a need to implement targeted insurance plans or subsidies to protect the vulnerable groups from incurring catastrophic health spending.

### 4.6. Limitations

Despite the advantages and reliability of WHO/HAI methodology for measuring access to medicines and DID analysis for policy evaluation [45,46], there are some limitations. First, the DiD analysis was limited due to the availability of price data from only two time points, and including additional time points would have enhanced the reliability and robustness of the findings. Nevertheless, the adjusted DiD model showed a statistically significant effect, with a higher R², indicating improved fit and greater precision by accounting for insulin characteristics. Subsequent studies should expand the dataset and include variables such as manufacturers’ policies and retailer behavior to provide a more detailed analysis.

Second, there could be variations compared to actual market prices before deregulation; however, the pre-deregulation (published) prices were obtained from a reliable and well-known source for retrospective study [19–21].

Third, we were only able to evaluate post-deregulation data for insulin availability in this study. Comparing it to our 2019 survey, however, gave helpful background information and, over time, showed significant trends.

Finally, the study did not include the prices and availability data from public sector hospitals; however, the medicines are provided free of cost in the public sector of Pakistan, and insulin is not provided in outpatient pharmacies. Therefore, patients predominantly obtain insulin from retail pharmacies, which were therefore exclusively analyzed to assess the impact of deregulation on insulin prices and affordability.

## 5. Conclusion

This study reveals the unintended consequences of Pakistan’s first-ever medicine price deregulation policy on insulin access, which include significant price hikes and decreased affordability, especially for underprivileged groups managing chronic conditions like diabetes. Although the goal of deregulation was to increase market efficiency, it highlights the need for a more focused strategy that protects access to life-saving medications in concentrated markets such as insulins. Regular price monitoring, promotion of locally produced insulins, and judicious price control mechanisms can increase competition and guarantee equitable access. To balance market dynamics with public health priorities, policymakers need to reevaluate the criteria for medicine price deregulation.

## Supporting information

S1 Table Median prices of insulin (10 mL 100 IU/mL) pre and post deregulation by duration of action in PKR. (DOCX)

S2 Table Affordability of insulin products (10 ml of 100 IU) before and after deregulation. (DOCX)

S3 Table Availability of insulin products by duration of action. (DOCX)

S4 Table City-wise number of insulin products across five retail pharmacies. (DOCX)

S1 Checklist Inclusivity in global research. (DOCX)

S1 Dataset Dataset used for analyses. (XLSX)
