# Supplementary material for: From medicine price control to deregulation: assessing policy effects on insulin access in Pakistan’s private pharmacies
Source: PLoS One. 2026 Mar 20;21(3):e0337151. doi: 10.1371/journal.pone.0337151 (PMC13004324; doi:10.1371/journal.pone.0337151)
Supplement: S1 Table — (DOCX) [file pone.0337151.s001.docx]

**S1 Table.** Median prices of insulin (10 mL 100IU/mL) pre and post deregulation by duration of action in PKR.

| Medicine Name | OB Pre | OB post | BS  (n) | BS Pre | BS post | Combined Pre | Combined Post | % Change |
| --- | --- | --- | --- | --- | --- | --- | --- | --- |
| Human insulin | **1035.0** | **1267.0** | **61** | **900.0** | **900.0** | **975.0** | **1230.5** | 26 |
| Short-acting human | 1035.0 | 1267.0 | 14 | 900.00 | 900.0 | 916.0 | 916.1 | 0 |
| Intermediate-acting human | 975.00 | 1267.0 | 29 | 916.0 | 916.1 | 975.0 | 1267.0 | 30 |
| Mixed human | 1241.0 | 1507.0 | 18 | 840.0 | 840.0 | 1241.00 | 1421.6 | 15 |
| Analogue insulin | **4288.6** | **5337.6** | **18** | **3640.0** | **3833.3** | **4288.6** | **5336.6** | 24 |
| Rapid-acting analogue | **4280.0** | **5146.4** | _ | _ | _ | **3746.6** | **4628.6** | 24 |
| Aspart | 4288.0 | 5146.5 | _ | _ | _ | 4448.0 | 5337.6 | 20 |
| Glulisine | 3533.3 | 4030.0 | _ | _ | _ | 3533.3 | 4030.0 | 14 |
| Lispro | 3745.6 | 4798.6 | _ | _ | _ | 3367.3 | 4622.0 | 28 |
| Long-acting analogue | **4717.4** | **5378.0** | **18** | **3640.0** | **3833.3** | **4717.43** | **5377.3** | 14 |
| Detemir | 4900.6 | 5583.3 | _ | _ | _ | 4900.66 | 5583.3 | 14 |
| Glargine | 4717.4 | 5378.0 | 18 | 3640.0 | 3833.3 | 4509.01 | 5377.3 | 19 |
| Mixed analogue | **9780** | **11149.2** | **_** | **_** | **_** | **9780** | **11149.2** | **14** |
| Aspart/degludec | 9780 | 11149.2 | _ | _ | _ | 9780 | 11149.2 | 14 |
