# Supplementary material for: From medicine price control to deregulation: assessing policy effects on insulin access in Pakistan’s private pharmacies
Source: PLoS One. 2026 Mar 20;21(3):e0337151. doi: 10.1371/journal.pone.0337151 (PMC13004324; doi:10.1371/journal.pone.0337151)
Supplement: S2 Table — (DOCX) [file pone.0337151.s002.docx]

**S2 Table.** Affordability of insulin products (10ml of 100IU) before and after deregulation.

| Medicine Name | NDWs pre-deregulation |  | NDWs post-deregulation |  | % Change in NDWs | |
| --- | --- | --- | --- | --- | --- | --- |
|  | Originator Brand | Biosimilar/ Generic | Originator Brand | Biosimilar/ Generic | Originator Brand | Biosimilar/ Generic |
| Human insulin | 0.97 | 0.84 | 1.19 | 0.84 | 22% | 0% |
| Short-acting human | 0.97 | 0.84 | 1.19 | 0.84 | 22% | 0% |
| Intermediate-acting human | 0.91 | 0.86 | 1.19 | 0.86 | 30% | 0% |
| Mixed human | 1.16 | 0.79 | 1.41 | 0.79 | 21% | 0% |
| Analogue insulin | 4.02 | 3.41 | 5.00 | 3.59 | 24% | 5% |
| Rapid-acting analogue | 3.51 | - | 4.34 | - | 24% | - |
| Aspart | 4.17 | - | 5.00 | - | 20% | - |
| Glulisine | 3.31 | - | 3.78 | - | 14% | - |
| Lispro | 3.16 | - | 4.33 | - | 37% | - |
| Long-acting analogue | 4.42 | 3.41 | 5.04 | 3.59 | 14% | 5% |
| Degludec | - | - | - | - | - | - |
| Detemir | 4.59 | - | 5.23 | - | 14% | - |
| Glargine | 4.42 | 3.41 | 5.04 | 3.59 | 14% | 5% |
| Mixed analogue | 9.17 | - | 10.45 | - | 20% | - |
| Aspart/degludec | 9.17 | - | 10.45 | - | 14% | - |

NDWs: Number of days’ wages required by a low-paid, unskilled government worker to purchase 10ml of 100IU insulin (approximate monthly treatment course).
