# Supplementary material for: From medicine price control to deregulation: assessing policy effects on insulin access in Pakistan’s private pharmacies
Source: PLoS One. 2026 Mar 20;21(3):e0337151. doi: 10.1371/journal.pone.0337151 (PMC13004324; doi:10.1371/journal.pone.0337151)
Supplement: S3 Table — (DOCX) [file pone.0337151.s003.docx]

**S3 Table. Availability of insulin products by duration of action.**

| Category | Insulin type | Originator  N (%) | Biosimilar  N (%) |
| --- | --- | --- | --- |
| Human insulin |  | **23.4 (78.0%)** | **16 (51.1%)** |
| Short-acting human |  |  |  |
|  | Regular (Soluble insulin) | 23 (76.6) | 15 (50.0) |
| Intermediate-acting human |  |  |  |
|  | Isophane (NPH) | 24 (80.0) | 16 (53.3) |
| Mixed human |  |  |  |
|  | Isophane/Regular (70/30) | 24 (80.0) | 16 (53.3) |
| Analogue insulin |  | **15 (48.8%)** | **2 (8.3%)** |
| Rapid-acting analogue |  |  |  |
|  | Aspart | 23 (76.6) | 0 |
|  | Glulisine | 4 (13.3) | 0 |
|  | Lispro | 16 (53.3) | 0 |
| Long-acting analogue |  |  |  |
|  | Detemir | 9 (30.0) | 0 |
|  | Glargine | 27(**90.0**) | 15 (50.0) |
|  | Degludec | 0 | 0 |
| Mixed analogue |  |  |  |
|  | Aspart/degludec | 9 (30.0) | 0 |

Where, N= Number of outlets where a particular insulin product was found.
