# Supplementary material for: From medicine price control to deregulation: assessing policy effects on insulin access in Pakistan’s private pharmacies
Source: PLoS One. 2026 Mar 20;21(3):e0337151. doi: 10.1371/journal.pone.0337151 (PMC13004324; doi:10.1371/journal.pone.0337151)
Supplement: S4 Table — (DOCX) [file pone.0337151.s004.docx]

**S4 Table.** City-wise number of insulin products across five retail pharmacies.

| City (Province/Territory) | Pharmacy 1 | Pharmacy 2 | Pharmacy 3 | Pharmacy 4 | Pharmacy 5 |
| --- | --- | --- | --- | --- | --- |
| Islamabad (Federal Capital) | 19 | 14 | 17 | 11 | 17 |
| Lahore (Punjab) | 23 | 24 | 26 | 14 | 13 |
| Faisalabad (Punjab) | 11 | 16 | 8 | 14 | 20 |
| Peshawar (Khyber Pakhtunkhwa) | 7 | 10 | 12 | 8 | 17 |
| Karachi (Sindh) | 23 | 6 | 12 | 5 | 16 |
| Quetta (Baluchistan) | 12 | 6 | 1 | 3 | 3 |
